# Omega-3 Polyunsaturated Fatty Acids Intake to Regulate *Helicobacter pylori*-Associated Gastric Diseases as Nonantimicrobial Dietary Approach

**DOI:** 10.1155/2015/712363

**Published:** 2015-08-03

**Authors:** Jong-Min Park, Migyeong Jeong, Eun-Hee Kim, Young-Min Han, Sung Hun Kwon, Ki-Baik Hahm

**Affiliations:** CHA Cancer Prevention Research Center, CHA Cancer Institute, CHA Bio Complex, Seongnam 463-400, Republic of Korea

## Abstract

Omega-3 polyunsaturated fatty acids (n-3 PUFAs), commonly eicosapentaenoic acid (EPA) and docosahexaenoic acid (DHA), have been acknowledged as essential long-chain fatty acids imposing either optimal health promotion or the rescuing from chronic inflammatory diseases such as atherosclerosis, fatty liver, and various inflammatory gastrointestinal diseases. Recent studies dealing with EPA and DHA have sparked highest interests because detailed molecular mechanisms had been documented with the identification of its receptor, G protein coupled receptor, and GPR120. In this review article, we have described clear evidences showing that n-3 PUFAs could reduce various *Helicobacter pylori*- (*H. pylori*-) associated gastric diseases and extended to play even cancer preventive outcomes including *H. pylori*-associated gastric cancer by influencing multiple targets, including proliferation, survival, angiogenesis, inflammation, and metastasis. Since our previous studies strongly concluded that nonantimicrobial dietary approach for reducing inflammation, for instance, application of phytoceuticals, probiotics, natural products including Korean red ginseng, and walnut plentiful of n-3 PUFAs, might be prerequisite step for preventing *H. pylori*-associated gastric cancer as well as facilitating the rejuvenation of precancerous atrophic gastritis, these beneficial lipids can restore or modify inflammation-associated lipid distortion and correction of altered lipid rafts to send right signaling to maintain healthy stomach even after chronic *H. pylori* infection.

## 1. Introduction

Gastric cancer is the fourth most common cancer and the second leading cause of mortality worldwide. Almost two-thirds of affected individuals will die from their disease. Gastric carcinogenesis has a multifactorial etiology.* Helicobacter pylori* (*H. pylori*) infection is the most important risk factor for both gastritis and gastric carcinoma [1]. Host genotype, bacterial virulence factors, and environmental conditions increase the risk of gastric cancer [2]. Gastric cancer develops due to* H. pylori* infection and chronic inflammation in a slow pace. It begins by an* H. pylori* infection that progresses to chronic active gastritis.* H. pylori* is adapted to survive in the acidic gastric environment. Bacterial adhesion to gastric epithelial cells induces inflammation that results in the recruitment of neutrophils followed by B and T lymphocytes, macrophages, and plasma cells. As a result, reactive oxygen and nitrogen species are produced, which are involved in gastric epithelial cell damage and carcinogenesis.* H. pylori* is considered to be the initiator of a chronic inflammatory response that summons bone marrow-derived cells to the gastric mucosa, thereby directly contributing to development of gastric cancer. Main virulence factors of* H. pylori* for the induction of mucosal inflammation are cytotoxin-associated gene (cag) pathogenicity island- (PAI-) encoded virulence factors, such as cytotoxin-associated antigen (CagA) protein, vacuolating toxin-A (VacA), blood group antigen-binding adhesion (BabA), and outer inflammatory protein (OipA). Under the influence of various environmental and host factors, chronic active gastritis may in turn evolve into atrophic gastritis and intestinal metaplasia. Metaplasia undergoes further genomic and phenotypic changes, resulting in gastric dysplasia and finally adenocarcinoma [3].

**Figure 1:**
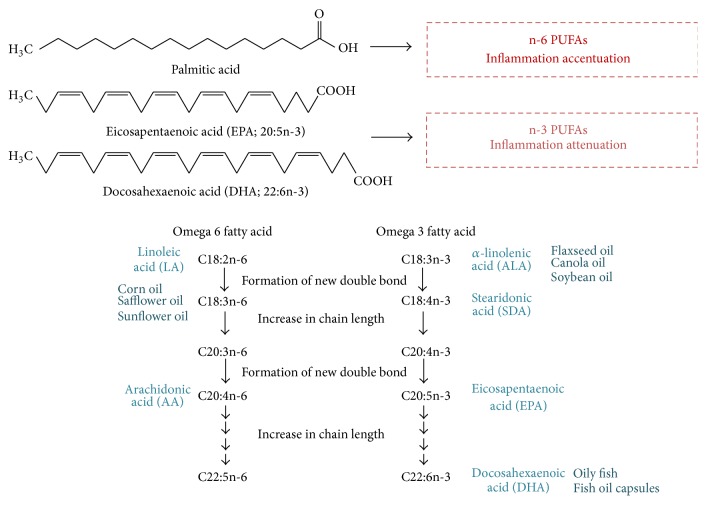
Essential n-3 PUFAs contributed to anti-inflammatory action based on their unsaturated bonds.

Omega-3 (n-3) polyunsaturated fatty acids [n-3 PUFAs, eicosapentaenoic acid (EPA 20:5n-3), and docosahexaenoic acid (DHA 22:6n-3)] are the long-chain PUFAs, which are essential fatty acids as they can be synthesized by mammals from other dietary precursors containing n-3 PUFAs. They are sufficiently found in fish. Fatty acids are key nutrients affecting early growth and development and preventing chronic disease in later life [4]. PUFAs that contain more than one carbon double bond are divided into two major classes, namely, n-6 and n-3 (Figure 1). Several lipid metabolites can be made from these PUFAs. Linoleic acid (LA 18:2n-6) is a representative n-6 PUFA, which is the precursor of arachidonic acid (AA 20:4n-6) that is involved in inflammation, inducing cardiovascular diseases, diabetes, cancer, and age-related diseases [4]. *α*-Linolenic acid (ALA), EPA, and DHA are important n-3 PUFAs that are required for human to remain healthy. Several studies suggest that n-3 PUFAs are capable of preventing diseases due to their antioxidant and anti-inflammatory characteristics. Particularly, n-3 PUFAs are shown to have protective effects against chronic inflammatory diseases such as cardiovascular diseases [5, 6], rheumatoid arthritis [7], diabetes [8], other autoimmune diseases [9, 10], and cancer [11, 12]. However, scant evidence exists for their role against* H. pylori* infection despite their known favorable effects.

Increased western-style fat consumption in the Eastern world (high in n-6 to n-3 PUFA ratio) is associated with the development of esophageal, breast, gastric, colon, and pancreatic and prostate cancers. Yet, n-3 PUFAs have multiple beneficial antitumor functions that are shown to change the malignant growth in a number of studies [13]. Studies on the fatty acid level in patients with bladder, pancreatic, lung, and esophageal cancer have shown low concentrations of plasma n-3 PUFAs, ranging from 55 to 88%, in comparison with healthy individuals [14–16]. Epidemiologic studies suggest that a high n-3 to n-6 PUFA ratio may be the optimal strategy to decrease cancer risk [17]. A solid epidemiologic study added the evidence that consumption of n-3 PUFAs appears to protect against the development of hepatocellular carcinoma even among patients with HBV and/or HCV infection [18]. A possible protective effect of dietary n-3 PUFAs against prostate cancer has been reported [19]. Fasano et al. [20] reviewed many* in vivo* and* in vitro* studies and found that n-3 PUFAs have antitumor properties against lung cancer. Then, how about the role of n-3 PUFAs against* H. pylori* infection?

## 2. Omega-3 Polyunsaturated Fatty Acids (n-3 PUFAs) and Inflammation

n-3 PUFAs are essential for health. They are widely studied for their roles in human health and disease [21]. Recently, they are shown to be effective in treating and preventing various diseases [22]. n-3 PUFAs have a therapeutic role against inflammatory diseases such as rheumatoid arthritis (RA), inflammatory bowel disease (IBD), asthma, and cardiovascular and neurodegenerative diseases [23–25]. A number of animal and human studies have provided convincing evidence for the anti-inflammatory effects of n-3 PUFAs. n-3 PUFAs are beneficial as a dietary supplement in RA by reducing the level of AA-derived eicosanoids and inflammatory cytokines, which include interleukin-1, interleukin-2, interleukin-6, and interleukin-8, as well as TNF-*α* and LTB_4_, promoting anti-inflammatory activities [26, 27]. Moreover, n-3 PUFAs have an NSAID-sparing effect in RA by decreasing pain and gastroduodenal damage [28, 29]. Dietary n-3 PUFA intake is associated with a decreased risk of developing Crohn's disease (CD) and ulcerative colitis (UC) by modulation of inflammatory events such as reducing LTB_4_, PGE_2_, and thromboxane B_2_ production [30–32]. n-3 PUFA administration has been shown to exert beneficial effects on animal model with induced lung inflammation or acute injury through decreasing the eosinophilic infiltration into the lung and improving the lung function [22, 33].* Fat-1* transgenic mice with elevated n-3 PUFAs levels have lower concentrations of proinflammatory cytokines and higher concentrations of protectin D1 and resolvin E1 in their lungs [34]. Fish oil with n-3 PUFAs decreases the incidence of atherosclerotic lesions and frequency of cardiac arrest contributing to a reduction in overall mortality in patients at risk of cardiovascular diseases [35–37]. n-3 PUFAs prevented the functional and anatomical changes in diabetic neuropathy, reduced oxidative damage, prevented learning disability in brain injury, and reduced the accumulation of *β*-amyloid in Alzheimer's disease [38–40].

Overall, supplementation of n-3 PUFAs directly reduces the production of inflammatory eicosanoids by replacing AA as an eicosanoid substrate and inhibiting AA metabolism by changing the cell membrane phospholipid fatty acid composition. n-3 PUFAs indirectly reduce the production of cytokines, reactive oxygen species (ROS), and adhesion molecules by altering the expression of inflammatory genes. This is achieved by intervening in the inflammatory signaling cascade, which include disruption of lipid rafts, activation of the anti-inflammatory transcription factor peroxisome proliferator-activated receptor *γ*, and binding to the G protein-coupled receptor GPR120 [23, 41]. Moreover, n-3 PUFAs can partially inhibit the inflammatory processes that involve leukocyte chemotaxis and T-helper 1 lymphocyte reactivity [41]. These properties suggest that n-3 PUFAs could have a therapeutic role in inflammatory diseases.

## 3. The Effect of n-3 PUFAs on Inflammation-Based Gastric Cancers

Most GI cancers including esophageal, stomach, and colorectal cancers have a natural history of multistep transition from precursor lesions to malignant lesions, over which inflammation, adenoma formation, and dysplastic changes prevail [42]. Therefore, GI cancers usually go through a premalignant lesion before development of the invasive cancer. Examples include Barrett's esophagus before esophageal cancer, chronic atrophic gastritis, and intestinal metaplasia before gastric cancer and adenoma or dysplasia in the setting of chronic ulcerative colitis before colon cancer. Since western diet contains disproportionally high amounts of n-6 PUFAs and low amounts of n-3 PUFAs (high n-6 to n-3 PUFA ratio), n-3 PUFAs-rich diets could become cancer preventive measures by affecting several stages of GI cancer development. Here, we introduce more carcinogenic detail regarding the interplay of inflammation and n-3 PUFAs intake.

Gastric cancer is the fourth most common cancer worldwide and almost two-thirds of affected individuals will die of their disease. Despite national efforts, 20 out of 100,000 Koreans die from gastric cancer. A number of studies investigating the association between n-3 PUFAs and gastric diseases suggest a protective effect for n-3 PUFAs in gastric cancer. Recently, Correia et al. [43] showed that DHA inhibits* H. pylori* growth* in vitro* and mice gastric mucosa colonization. It has been proposed that PUFAs hold an inhibitory effect on bacterial growth via disruption of cell membrane leading to bacteria lysis [44]. Mohammed et al. [45] showed that n-3 PUFAs reduce iodoacetamide-induced gastritis in rats by decreasing malondialdehyde (MDA), gastrin and nitric oxide (NO), and normalizing mucosal glutathione. Erythrocyte composition of DHA is found to be negatively associated with the risk of well-differentiated gastric adenocarcinoma [46]. n-3 PUFAs-rich diet delayed tumor growth in a mouse xenograft model of gastric cancer [47]. Another* in vitro* study showed that n-3 PUFAs inhibit macrophage-enhanced gastric cancer cell migration and attenuate matrix metalloproteinase- (MMP-) 10 expression through ERK and STAT3 phosphorylation [48] and inhibit the growth of human gastric carcinoma via apoptosis [49]. Moreover, n-3 PUFAs are beneficial for preventing oxidative stress-induced apoptosis by inhibiting apoptotic gene expression and DNA fragmentation of gastric epithelial cells [50]. DHA induces apoptosis of gastric cancer cells by inducing the expression of apoptotic genes [51]. Although a large body of literature spanning numerous cohorts from many countries with different demographic characteristics does not provide evidence to suggest a significant association between n-3 PUFAs and the incidence of stomach cancer [52], precise studies are required to investigate the antitumor properties of n-3 PUFAs in the stomach.

## 4. Molecular Mechanisms of n-3 PUFAs Effect against* H. pylori* Infection

### 4.1. Attenuation of* H. pylori*-Associated Inflammation

*H. pylori* is clever as it changes n-6 PUFAs metabolism to foster gastric environment or to enhance gastric inflammation. Nakaya et al. [53] studied the formation of n-6 PUFAs from LA in rat gastric mucosal cells after* H. pylori* infection. They found that addition of LA leads to an increase in the composition of AA, LA, and PGE_2_. Cyclooxygenase-2 (COX-2) expression is induced in* RGM-1* cells by addition of LA.* H. pylori* culture broth filtrates had decreased LA and increased AA compositions. Moreover, after incubation with* H. pylori* culture filtrates, PGE_2_ concentrations were higher than controls. Thus,* H. pylori* infection can enhance PGE_2_ synthesis and accelerate n-6 PUFAs metabolism in gastric mucosal cells, which can make the gastric mucosal barrier more fragile. On the other hand, since essential dietary fatty acids bestow gastroduodenal mucosal protection [54], n-3 PUFAs can provide efficient gastroprotection against* H. pylori*, drug-induced and stress-induced gastric mucosal damages [55]. These effects were first observed in 1987 when the link between low levels of PUFAs and* H. pylori*-associated duodenal ulcer was shown in an interventional dietary study [56]. Recent studies have shown anti-inflammation potential propertied for n-3 PUFAs [57, 58]. n-3 PUFAs suppressed the activation of EGFR, PKC *δ*, MAPK, NF-*κ*B, and AP-1 in* H. pylori*-infected gastric epithelial* AGS* cells. n-3 PUFAs are beneficial for prevention of* H. pylori*-associated gastric inflammation by inhibiting proinflammatory IL-8 expression [57].* AGS* cells that were infected with DHA pretreated* H. pylori* showed a 3-fold reduction in IL-8 production and a decrease in COX-2 and inducible nitric oxide synthase (iNOS) [58]. However, the other side of the n-3 PUFAs coin is shown in gastrointestinal inflammations, where worse effects are reported with n-PUFAs despite the above-mentioned positive effects. For example, Woodworth et al. [59] and Butler and Yu [60] studied the effect of dietary fish oil enriched with DHA on reduction of experimentally induced colitis and colon cancer risk. In their experiments, they infected* Smad*3 null mice with* H*.* hepaticus* to induce colitis. They observed mild colitis 4 weeks after infection, but paradoxically, mice fed with isocaloric diets modified to include corn oil, sunflower oil, or DHA (as the fatty acid source) developed severe colitis and adenocarcinoma with DHA after 8 weeks. They concluded that DHA-fed mice may be less equipped to mount a successful response to* H. hepaticus* infection (increasing colon cancer risk), supporting the need to establish a tolerable upper limit for DHA intake particularly in the setting of chronic inflammation. In another study, DHA-fed* Smad*3 knockout mice had significantly higher levels of plasma IL-5, IL-13, and IL-9 (Th2-biasing cytokines) and cecal IgA compared with control, leading to the emerging concept that fish oil might enhance B cell function* in vivo* to aggravate an existing inflammatory response [61]. A proposed molecular mechanism by which DHA exerts its effects is modification of lipid raft organization [62]. Regarding the discrepancy in the efficacy of n-3 PUFAs, Rockett et al. [63] demonstrated that a physiologically relevant dose of n-3 PUFAs increases the size of B cell rafts, GM1 surface expression, and membrane molecular order upon cross-linking. Increasing lipid raft size with fish oil was accompanied by changes in innate and adaptive function. Other factors should also be exploited to use n-3 PUFAs as a safer rescue agent against* H. pylori* infection in future.

### 4.2. Antimicrobial Activity against* H. pylori*: A Direct Effect on the Bacteria

Eradication of* H. pylori* with a proton pump inhibitor-based triple therapy is currently the treatment of choice for* H. pylori* infection. However, it has a success rate of 80–90%. Treatment failure and contraindications in some patients are common. Furthermore, rapidly emerging drug resistance in* H. pylori* strains during treatment with different antibiotics is a major obstacle for successful eradication. Due to the prevalence of antibiotic-resistant* H. pylori* strains, there is an increasing effort in seeking other safe and effective compounds that inhibit* H. pylori* growth. Recent studies have shown that n-3 PUFAs have anti-*H. pylori* potentials. PUFAs significantly inhibit* H. pylori*'s growth [44]. A very recent* in vitro* and* in vivo* investigation [43] clearly showed that n-3 PUFAs inhibit* H. pylori* growth* in vitro* and its colonization in mice gastric mucosa* in vivo*. This study is implicated in therapy and alleviation of* H. pylori*-induced inflammation. Since drug-resistant* H. pylori* strains and noncompliance to therapy are the major causes of* H. pylori* eradication failure, n-3 PUFAs could be used to lower the recurrence rate of infection in combination with standard triple therapy. This can prohibit* H. pylori*'s ability to colonize the mouse stomach. DHA has also been shown to decrease* H. pylori*'s growth and associated inflammation. Anti-*H. pylori* effects of DHA are associated with changes in bacterial morphology, metabolism, and alteration of the composition of outer membrane proteins, which ultimately reduces the adhesion of bacteria and the burden of* H. pylori*-related inflammation [58]. In contrast to the* in vitro* bacteriostatic and bactericidal efficacy of n-3 PUFAs, Meier et al. [64] showed that fish oil is less effective than metronidazole, in combination with pantoprazole and clarithromycin, for* H. pylori* eradication.

### 4.3. Imposing Restorative Mechanism and Reducing* H. pylori*-Induced Cytotoxicity

Regeneration-promoting role of n-3 PUFAs is well acknowledged in several medical fields, namely, liver regeneration [65], wound healing [66], bone preservation [67, 68], burn [69], endothelial regeneration [70], cellular resolution of inflammation [71], recovery from nerve injury [72], and regeneration from previous intestine surgery [73]. n-3 PUFAs are shown to prevent acute liver failure and promote liver regeneration in rats by protecting the structure of the sinusoidal endothelial cells (SEC) in the acute phase after hepatectomy along with phosphorylation of the STAT3 and Akt [74]. A large number of animal and human studies have reported a positive n-3 PUFAs effect on increased bone formation, reduced bone resorption, and protection from osteoporosis [68]. Improvement in bone health is achieved by attenuation of mediators of osteoclastogenesis, namely, PGE2, COX-2, IL1-*β*, TNF*α*, and NF-*κ*B, in particular via the E (EPA derived) and D (DHA derived) series of resolvins. Eicosapentaenoic acid-derived resolvin E1 (RvE1) enhances resolution of inflammation, prevents bone loss, and induces bone regeneration [67]. RvE1 modulates osteoclast differentiation and bone remodeling by direct actions on bone. It rescues osteoprotegerin (OPG) production and restores a favorable NF-*κ*B ligand/OPG receptor-activator ratio along with known anti-inflammatory and proresolving actions. RvD2 effectively prevents thrombosis of the deep dermal vascular network (DDVN) in early phases of burn injury. Therefore, it reduces secondary tissue damage and promotes long-term survival of deep dermal components after the initial thermal insult [69]. Alexander et al. have shown improved protein synthesis after burn injury in rats receiving n-3 PUFAs supplementation [75]. Interestingly, in a model of corneal nerve regeneration after surgery, it has been shown that DHA has strong proregenerative effects in combination with pigment epithelial growth factor. The authors hypothesized that this may involve formation of neuroprotectin D1 (a DHA metabolite) [76]. Dietary supplementation with n-3 PUFAs improved colonic anastomoses healing [73]. n-3 PUFAs enhance the colonic wound healing in a rat model. Finally, n-3 PUFAs may prompt faster resolution of inflammation within the wound microenvironment, which leads to facilitated regeneration and reepithelialization. A small randomized controlled trial evaluated a formula supplemented with fish oil in patients with pressure ulcers and noted decreased progression of pressure ulcers in those receiving fish oil supplementation [77].

There is growing evidence that the diverse biological roles of n-3 PUFAs contribute to their regenerative actions against chronic inflammatory disease. This could effectively help resolve the inflammation and promote a transition from the inflammatory to the proliferative and remodeling phases of wound healing [78]. n-3 PUFAs can be incorporated into membrane phospholipids, which causes reduced membrane fluidity. It could be associated with lipid raft assembly and function. Lipid rafts are cholesterol-rich microdomains at the host cell surface and are required for NF-*κ*B-dependent responses to* H. pylori*. Recently, several studies have suggested that n-3 PUFAs can be converted into bioactive mediators, including resolvins, which exert inflammation-resolving properties via counterregulation of lipid mediators including proinflammatory leukotriene (LTs) and prostaglandin (PGs). Thus, our group investigated long-term treatment of n-3 PUFAs in an* H. pylori*-infected animal model and found that n-3 PUFAs administration ameliorated* H. pylori*-induced gastric inflammation and atrophic gastritis. It also reduced the incidence of* H. pylori*-associated gastric carcinogenesis (unpublished data). We could be the first group to document the rejuvenating action of n-3 PUFAs on* H. pylori*-associated atrophic changes in stomach. As the use of n-3 PUFAs for treatment of* H. pylori*-induced GI disorders is rapidly moving into clinical settings with more studies explaining the mechanism of action, detailed randomized controlled trials are required to obtain strong evidence for the incorporation n-3 PUFAs into the therapeutic armamentarium in near future.

### 4.4. *H. pylori*-Induced Oxidative Stress and the Scavenging Action of n-3 PUFAs

Although limited reports are available on the antioxidative action of n-3 PUFAs against* H. pylori* infection, this antioxidative feature is well known in cardiovascular and neurological fields. A very recent high impact evidence came from Lluís et al. [79] showing that a diet of 1 : 1 ratio of EPA/DHA improves many oxidative stress parameters like superoxide dismutase (SOD) and glutathione peroxidase (GPx) in erythrocytes, plasma antioxidant capacity, and cardiovascular risk factors (glycated hemoglobin) relative to the other diets. Avramovic et al. [80] showed beneficial effects of n-3 PUFAs on the oxidative stress in brain tissue, improving neurogenesis and neuroplasticity [81]. Regarding antioxidative effects of n-3 PUFAs against* H. pylori* infection, we investigated the direct scavenging action of EPA using electron spin resonance and found significant antioxidative actions against hydroxyl and superoxide radicals.

### 4.5. Proposed Mechanisms of n-3 PUFAs Cancer Prevention against* H. pylori*-Associated Carcinogenesis

n-3 PUFAs can suppress the anti-inflammatory-related function of T lymphocytes and antigen presenting cells (APCs). The mechanism by which DHA modifies lymphocyte function is changing the organization of sphingolipid/cholesterol lipid raft membrane domains. Two contradictory models have been proposed to explain how DHA exerts its effects by changing the raft organization. One is the cellular model, which proposed that DHA is directly incorporated into the lipid rafts and changes the protein activity to suppress the lymphocytic functions. The other suggests modification of lymphocytic function by formation of nanometer scale DHA-rich domains, which disrupts the optimal raft-dependent clustering of proteins necessary for initial signaling. A biophysical DHA-containing phospholipid membrane model showed that unique nonraft membrane domains are formed that are organizationally distinct from lipid rafts. This alters the conformation and/or lateral organization of lymphocyte proteins [82]. Based on n-3 PUFAs effects on lymphocytes and modulation of inflammation, n-3 PUFAs, especially EPA or DHA, are shown to have multiple antigastric-tumor actions. Our current knowledge of the antitumor activity of n-3 PUFAs has been comprehensively reviewed elsewhere [12, 19, 83]. Recently, Stephenson et al. [13] have reviewed more recent underlying mechanisms of antitumor properties of n-3 PUFAs. n-3 PUFAs inhibit growth signal transduction. They appear to downregulate epidermal growth factor receptor (EGFR), protein kinase C (PKC),* Ras*, NF-*κ*B, and insulin-like growth factor (IGF), which are important cell-signaling mediators often found to be elevated in cancer. n-3 PUFAs also induce cancer cell apoptosis via modulation of peroxisome proliferator-activated receptors (PPARs), Bcl-2 family, and NF-*κ*B cell signaling. They decrease sprouting angiogenesis by suppressing vascular endothelial growth factor- (VEGF-) and platelet derived growth factor- (PDGF-) stimulated endothelial cell proliferation, migration and tube formation through inhibition of MMPs via NO production, and NF-*κ*B and *β*-catenin cell signaling. Moreover, n-3 PUFAs decrease cell-cell adhesion via downregulation of Rho-GTPase (inhibits cytoskeleton reorganization) and reduction in intercellular adhesion molecule- (ICAM-) 1 and vascular cell adhesion molecule- (VCAM-) 1 expression.

Cockbain et al. [12] have proposed four main antitumor actions for n-3 PUFAs, namely, (1) modulation of COX activity, (2) alteration of membrane dynamics and cell surface receptor function, (3) increase in cellular oxidative stress leading to cytotoxicity, and (4) enhancement of anti-inflammatory lipid mediators. In mechanistic details, n-3 PUFAs can act as alternative substrates for COX-2 (instead of AA) leading to a reduction in formation of protumorigenic “2-series” PGs (PGE_2_) in several cell types. They also bind the substrate channel of COX-2 and inhibit COX-2 activity. In addition, incorporation of n-3 PUFAs into the cell membrane alters the fluidity, structure, and/or function of lipid rafts or caveolae. Localization of cell surface receptors such as G protein-coupled receptors (GPCRs), toll-like receptors (TLRs), and epidermal growth factor receptor (EGFR) in lipid rafts is believed to be crucial for downstream receptor signaling, which controls proliferation and apoptosis.

Rockett et al. [84] showed that membrane raft organization is more sensitive to disruption by n-3 PUFAs than the nonraft organization. n-3 PUFAs can also have an antitumor effect via alteration of the cellular redox state. They can increase ROS due to being highly peroxidizable. Therefore, n-3 PUFAs can induce cancer cell apoptosis via elevation of intracellular ROS levels. Lastly, n-3 PUFAs can be metabolized into novel anti-inflammatory lipid mediators including resolvins, protectins, and maresins. Resolvins exhibit antineoplastic activity via anti-inflammatory and inflammation resolution features in animal models of acute inflammation. Nuclear factor erythroid 2-related factor 2 (Nrf2) is a redox-sensitive master regulatory transcriptional factor that plays an important protective role in cells by regulating cellular redox balance [85]. Recently, it was reported that n-3 PUFAs can activate Nrf2 and induced Nrf2-directed gene expression [86, 87]. They can suppress lipopolysaccharide-induced inflammation through induction of Nrf2 expression [88]. Last but not least, n-3 PUFAs significantly reduce oxidative stress-induced endothelial cell Ca^++^ influx. This effect might be associated, at least in part, with altered lipid composition of membrane lipid rafts [89].

## 5. Dietary Walnuts Intake as Source of n-3 PUFAs to Rejuvenate* Helicobacter pylori*-Associated Atrophic Gastritis as well as to Augment* H. pylori* Eradication Rate

It has been reported that Asian dietary pattern and* H. pylori* infection are typically associated with increased risk of gastric cancer.* H. pylori*, a Gram-negative bacterial pathogen that infects approximately 50% of the world's population, provokes chronic gastric inflammation, which is considered to be a major risk factor for development of gastric and duodenal ulcers, mucosa-associated lymphoid tissue lymphoma (MALToma), and gastric cancer [2]. Despite the existing debates, there is enough evidence that* H. pylori* infection is strongly associated with gastric cancer, for which it is defined as a class I carcinogen by IARC [90–92]. Mechanistically, gastric epithelial cells respond to* H. pylori* infection by upregulating the expression of proinflammatory genes, which includes the upregulation of COX-2, iNOS, and IL-8 [93, 94]. With perpetuating gastric inflammation and oxidative stress,* H. pylori* infection gives rise to significant DNA damage, apoptosis, and cell cycle dysregulation, all of which are closely associated with significant oncogenic insults on infected mucosa [95, 96]. Since gastric cancer is a multistep and multifactorial disease and not every individual infected with* H. pylori* develops gastric cancer, population-wide eradication strategies are not generally considered as the preventive measures for gastric cancer [97].

*H. pylori*-related gastric diseases are now considered as infectious diseases and are treated with antibiotic regimens, which are often composed of two antibiotics (amoxicillin and clarithromycin) and a proton pump inhibitor [97, 98]. However, this treatment can fail for several reasons, which include insufficient antibiotic concentration due to mutation-acquired resistance, limited antibiotic efficacy in a low gastric pH, and insufficient antibiotic concentration due to a high bacterial load [99]. In addition, existence of viable bacteria in dormant forms that are not accessible to antibiotics and antibiotic-related damage to the mucosal immunity can also be observed. Seeking alternatives to solve the problems of the expense, lower compliance and antibiotic resistance has stretched to nonantimicrobial approaches, which include supplementation with probiotics and other nutrients.

Fresh antioxidant fruits, vegetables, and certain micronutrients such as selenium and vitamin C can reduce the risk of infection. A food that can inhibit* H. pylori* viability, colonization, and infection can also reduce the risk of cancer. Korean red ginseng, licorice extracts, S-allyl cysteine (SAC) from garlic, probiotics like* L. plantarum* or* Bifidobacillus,* and n-3 PUFAs are shown to be effective antibacterials and antimutagenics along with rejuvenating activities in* H. pylori* infection. Our group extended the supplementation of effective phytoceuticals or phytonutrients to achieve higher eradication rates and possibly prevent the* H. pylori*-associated gastric cancer [43, 44, 100–102].

Mere eradication yielded limited benefit in some clinical diseases but supplementation with natural products or phytoceuticals rendered higher eradication rates. It is also associated with fewer side effects compared to the triple regimen, higher levels of anti-inflammatory cytokines, and some restorative actions. Our studies showed that SAC supplementation could lead to efficient reduction in inflammation due to potent demethylating mechanism of HDAC inhibition. It can also enforce the regenerative activities along with its antimutagenic action.

Natural phytochemicals with antioxidant, anti-inflammatory, and anticarcinogenic properties that regulate or target specific molecules in gastric carcinogenesis can increase the efficacy of* H. pylori* eradication as an effort to prevent gastric cancer. Nuts and seeds, particularly walnut, are nutritionally dense and rich in vitamin E and n-3 PUFAs. They are an ample source of dietary fiber, B vitamin, and essential mineral such as magnesium, copper, manganese, calcium, and potassium as well as monounsaturated and polyunsaturated fats, which can potentially lower the LDL cholesterol [103]. Potential health benefits, which have not been scientifically validated, include improved complexion and decreased risk of cancer [104, 105].

The fundamental basis of walnuts treatment in* H. pylori *infection is suppression of inflammation and/or inhibition of pathogenic bacteria. There is the possibility that the enhancement of negative regulators of inflammation-associated cytokine by walnuts could also contribute to* H. pylori* eradication. Sliced walnuts contain biotin, vitamin R, manganese, copper, vitamin B12, phosphorus, magnesium, molybdenum, and fiber. Walnut is also high in monounsaturated fats, the same type of health-promoting fat found in olive oil, which have been associated with reduced risk of heart disease. In addition to the healthy fats and vitamin E, a quarter-cup of walnuts contains 62 mg of magnesium and 162 mg of potassium. These nutritional facts can shed light on the possible beneficial effects of walnuts in* H. pylori* treatment.

We postulate that high walnut's n-3 PUFAs and vitamin E contents help the treatment and eradication of* H. pylori* infection.* H. pylori* creates a microenvironment to protect itself from gastric acid and host defense systems. It increases the oxidative stress in its colonized areas. It is shown that ROS levels are increased in patients with* H. pylori* infection and that ROS levels are decreased following* H. pylori *eradication. Vitamin E impairs lipid preoxidation pathways and exerts an antioxidant effect. It is also shown that vitamin E concentration is low in the gastric fluid and mucosa of* H. pylori*-infected patients and high after* H. pylori* eradication. These facts suggest a possible benefit from vitamin E supplementation in* H. pylori* infection.

In animal models, short-term therapy with vitamin E is shown to have restorative effects on* H. pylori* gastritis. It is also shown to have protective effects on* H. pylori*-induced mucosal injury by preventing the accumulation of activated neutrophils. In conclusion, therapeutic doses of vitamin E decrease the intensity of* H. pylori *infection and neutrophil activity in the antrum. These effects on the bacterial microenvironment could be the reason for the supplementation with antibiotics to increase the eradication rate [106]. We have solid evidence that n-3 PUFAs ameliorated* H. pylori*-induced gastritis and significantly prevented the gastric cancer. However, some studies showed that magnesium affects the outcome.

## 6. Conclusion

**Figure 2:**
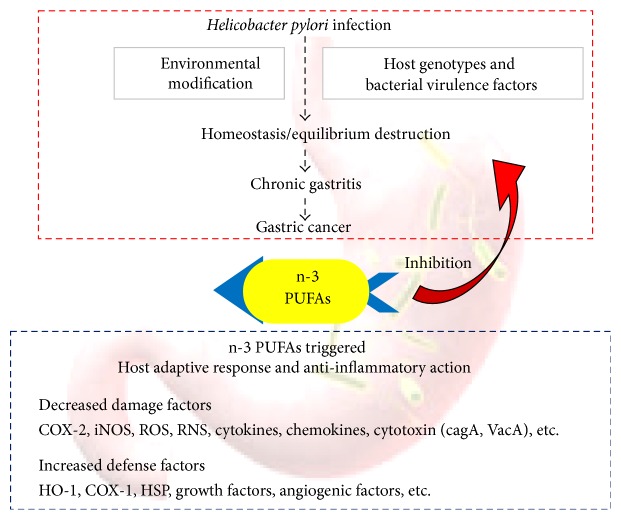
Host adoptive and antimutagenic response enhanced with n-3 PUFAs in* H. pylori* infection.

Although* H. pylori* is defined as a class 1 carcinogen by IARC, debate exists on whether the eradication can prevent the associated gastric cancer. Furthermore, host genotype, bacterial virulence factors, and environmental factors can affect the outcome after* H. pylori *infection. Host adaptive responses can modify the carcinogenic influence of* H. pylori* infection (Figure 2). n-3 PUFAs are known to be antioxidant, anti-inflammation, and anticancer. Recently, several investigators have examined the anti-inflammatory, antimicrobial, and rejuvenating effects of n-3 PUFAs on* H. pylori*-induced gastric diseases (Figure 2). There is accumulating evidence that suggests higher consumption of dietary n-3 PUFAs is associated with lower risk of GI cancer development in animal models and human. Recent studies suggest that endogenous n-3 PUFAs delay the progression of stomach cancer and elevating n-3 PUFAs could be an important strategy to delay/prevent gastrointestinal cancer in high-risk patients. This effect is bestowed via several mechanisms mediating cancer prevention such as anti-inflammatory and rejuvenating effects of n-3 PUFAs against carcinogenic* H. pylori* infection. Despite the orchestrated mechanisms of action, maintenance of optimal cellular levels of n-3 PUFAs can be the royalty gatekeeper of* H. pylori*-associated gastric inflammation and cancer. Supplementation with n-3 PUFAs in combination with other antitumor agents can possibly improve the efficacy of GI cancer prevention. Large-scale translational and comprehensive mechanism studies are required to demonstrate the preventive or therapeutic effects of n-3 PUFAs against* H. pylori*-induced gastric diseases. Clinical trials are prerequisites of the decision on the role of chemopreventive agents in* H. pylori*-associated gastric cancer. A long-term study that shows the preventive effect of n-3 PUFAs against gastritis and* H. pylori*-induced tumorigenesis will be a prime example of cancer prevention by food.
